# Differential effects of heat-not-burn and conventional cigarettes on coronary flow, myocardial and vascular function

**DOI:** 10.1038/s41598-021-91245-9

**Published:** 2021-06-03

**Authors:** Ignatios Ikonomidis, Dimitrios Vlastos, Gavriela Kostelli, Kallirhoe Kourea, Konstantinos Katogiannis, Maria Tsoumani, John Parissis, Ioanna Andreadou, Dimitrios Alexopoulos

**Affiliations:** 1 2nd Cardiology Department, Attikon Hospital, National and Kapodistrian University of Athens, Rimini 1, Haidari, 12462 Athens, Greece; 2 Department of Cardiac Surgery, Royal Brompton Hospital, Imperial College, London, UK; 3 Department of Pharmaceutical Chemistry, National and Kapodistrian University of Athens, School of Pharmacy, Athens, Greece

**Keywords:** Cardiology, Health care, Medical research, Pathogenesis, Risk factors

## Abstract

We compared the effects of Heat-not-Burn cigarette (HNBC) to those of tobacco cigarette (Tcig), on myocardial, coronary and arterial function as well as on oxidative stress and platelet activation in 75 smokers. In the acute study, 50 smokers were randomised into smoking a single Tcig or a HNBC and after 60 min were crossed-over to the alternate smoking. For chronic phase, 50 smokers were switched to HNBC and were compared with an external group of 25 Tcig smokers before and after 1 month. Exhaled carbon monoxide (CO), pulse wave velocity (PWV), malondialdehyde (MDA) and thromboxane B2 (TxB2) were assessed in the acute and chronic study. Global longitudinal strain (GLS), myocardial work index (GWI), wasted myocardial work (GWW), coronary flow reserve (CFR), total arterial compliance (TAC) and flow-mediated dilation (FMD) were assessed in the chronic study. Acute HNBC smoking caused a smaller increase of PWV than Tcig (change 1.1 vs 0.54 m/s, p < 0.05) without change in CO and biomarkers in contrast to Tcig. Compared to Tcig, switching to HNBC for 1-month improved CO, FMD, CFR, TAC, GLS, GWW, MDA, TxB2 (differences 10.42 ppm, 4.3%, 0.98, 1.8 mL/mmHg, 2.35%, 19.72 mmHg%, 0.38 nmol/L and 45 pg/mL respectively, p < 0.05). HNBCs exert a less detrimental effect on vascular and cardiac function than tobacco cigarettes.

**Trial registration** Registered on https://clinicaltrials.gov/ (NCT03452124, 02/03/2018).

## Introduction

Smoking constitutes a major modifiable risk factor for cardiovascular disease ^1^. The pathophysiological mechanisms underlying its deleterious effects include arterial elasticity ^2^ and myocardial deformation impairment ^3^, endothelial dysfunction ^4^, oxidative stress accentuation ^2^, and platelet activation enhancement ^5^. Carotid-femoral pulse wave velocity (cfPWV) measurement allows a reproducible, non-invasive quantification of arterial stiffness ^6^ with diagnostic ^7^ and prognostic value ^8^. In addition, total arterial compliance (TAC) represents a pulsatile component of left ventricular (LV) afterload and quantifies the compliance of the entire arterial system, with significant physiological and prognostic implications ^9^. Flow mediated dilation (FMD) of the brachial artery ^10^ evaluates endothelial function and predicts cardiovascular mortality. Every 1% improvement in FMD has been associated with 13% reduction of the risk for cardiovascular events ^11^. Coronary flow reserve (CFR) as assessed by echocardiography is marker of coronary microcirculatory function with predictive value for adverse cardiovascular outcome ^12^. Importantly, CFR may predict mortality and risk for myocardial infarction (MI) in patients with normal or near-normal coronary arteries and preserved regional and global left ventricular function both at rest and during stress ^13^. In this respect, smoking has been demonstrated to reduce CFR by 21% in asymptomatic smokers without coronary artery disease ^14^. Global longitudinal strain (GLS) computation by speckle tracking echocardiography is a standardized modality of myocardial deformation assessment ^15^. Myocardial work index (MW) is a novel index of the ventricular-arterial interaction which combines longitudinal myocardial deformation with dynamic non-invasive LV pressure measurements creating pressure–strain loops. Thus, this index minimizes pitfalls of myocardial strain examination that derive from its load-dependent nature ^16^. Regarding oxidative stress burden, plasma malondialdehyde (MDA) ^17^ and protein carbonyls (PC) ^18^ concentration serve as widely used biomarkers, while plasma thromboxane B2 (TxB2) levels are associated with increased platelet activation ^19^.

More than 5600 chemical components have been identified in traditional tobacco cigarettes, many of which harmfully affect the cardiovascular system ^20^. In this respect, the development of potential reduced exposure products (PREPs) has been suggested as a means to reduce the adverse effects associated with the use of tobacco products ^21^. Although first generation PREP use was, indeed, associated with reduced biomarkers of exposure (BoE) levels, neither oxidative stress burden nor markers of inflammation were improved ^22^. Heat-not-burn cigarette (HNBC) has been developed as a new-generation non-combustible PREP ^23^. Early evidence suggests that its use results in a significantly reduced BoE levels in comparison with traditional combustible cigarettes ^24–31^; however, this has not been consistently associated with cardiovascular function benefits. On the one hand, evidence has arisen that HNBC acutely impairs oxidative stress equilibrium, platelet activation, endothelial function, and peripheral blood pressure to a lesser extent than traditional smoking ^32^. On the other hand, in rat models, exposure to HNBC aerosol was as detrimental to FMD as cigarette smoke ^33^. Thus, the effects of HNBC use on cardiovascular function have not been fully defined.

The aim of this study was to investigate the effects of HNBC on endothelial function, arterial stiffness, myocardial deformation, oxidative stress, and platelet activation both in an acute context and after 1 month of switching to HNBC smoking, in comparison with traditional tobacco cigarette.

## Results

### Study population

The mean age of the 50 participants was 48 ± 5 years, 53% were female, and reported smoking 27 ± 9 cigarettes per day per individual with a smoking history of 38 ± 18 pack-years. Tobacco smokers, used as controls, had a mean age of 46 ± 14 years, 53% were female and reported smoking 26 ± 8 cigarettes per day per individual with a smoking history of 37 ± 19 pack-years. All subjects had similar baseline characteristics in terms of arterial stiffness, myocardial deformation, oxidative stress, and platelet activation status (Table 1).

**Table 1:** Characteristics of the study cohort.

	Intervention N = 50	Control N = 25	p-value
Age (years)	48 ± 9	46 ± 11	0.7
Sex (female)	27 (53%)	14 (53%)	0.6
SBP (mmHg)	125 ± 15	121 ± 13	0.7
DBP (mmHg)	79 ± 10	76 ± 10	0.6
Heart rate (bpm)	66 ± 9	67 ± 8	0.8
BMI (kg/m^2^)	27.2 ± 5.1	27.3 ± 5.2	0.9
CO (ppm)	14.9 ± 7.4	15.8 ± 4.9	0.7
Pack-years	38 ± 18	37 ± 19	0.3
LDL-cholesterol (mg/dl)	114 ± 22	118 ± 19	0.5
CRP-hs (mg/dl)	1.8 ± 0.4	1.9 ± 0.5	0.4

*SBP* systolic blood pressure, *DBP* diastolic blood pressure, *BMI* body mass index, *BP* blood pressure, *CO* exhaled carbon monoxide, *CRP-hs* C-reactive protein highly sensitive, values are mean ± SD.

During the chronic study, only 3 of the 50 (6%) participants reported simultaneous use of IQOS with traditional cigarettes (< 5 per day). The remaining 47 participants successfully refrained from traditional cigarette smoking and used HNBC exclusively during the 1-month follow-up period (17 ± 6 heets per day per individual). No side-effects were reported. In the control group, smokers used 28 ± 8 Tcig per day per individual during one month.

### Acute study

#### Vascular function

**Figure 1:**
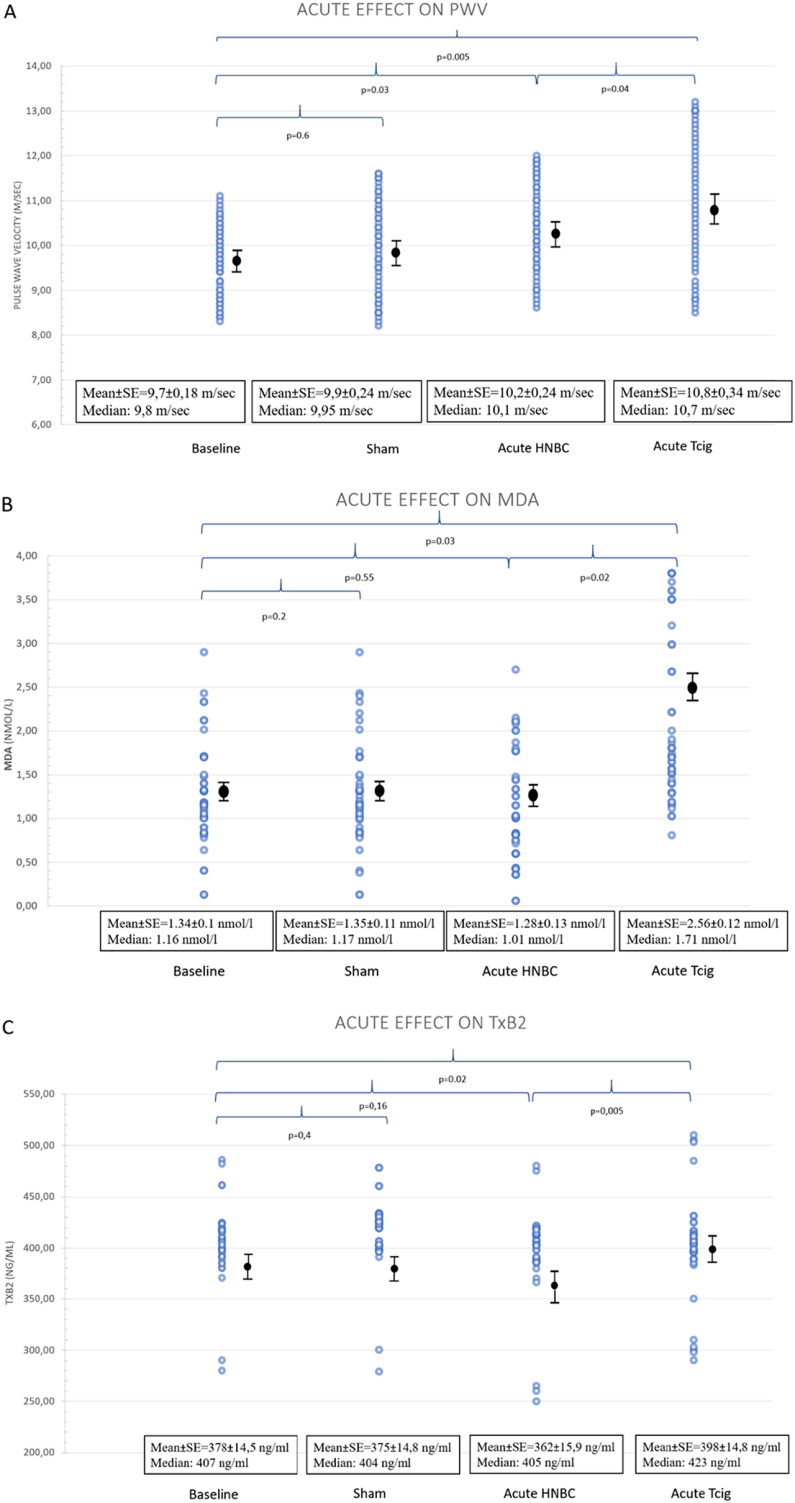
Acute effects of heat-not-burn puffing versus tobacco cigarette smoking. Comparison between the acute effects of heat-not-burn versus tobacco cigarette smoking on (**A**) arterial stiffness, (**B**) oxidative stress burden, and (**C**) platelet activation status. HBNC showed a smaller increase of PWV than tobacco cigarette. All biomarkers are impaired following Tcig smoking, in contrast with HNBC puffing. *PWV* carotid-femoral pulse wave velocity, *HNBC* heat-not-burn cigarette, *MDA* malondialdehyde, *Tcig* tobacco cigarette, *TxB2* thromboxane B2, *SE* Standard Error.

During the acute study, PWV was increased after HBNC and Tcig smoking compared to baseline (Table 2, p = 0.04 and p = 0.005 respectively). However, Tcig smoking resulted in a greater increase of PWV than HNBC puffing (mean change 1.11 m/s; 95% CI 0.35–1.18, p = 0.005 vs 0.54 m/s; 95% CI 0.05–1.04, p = 0.03 respectively, difference = 0.57 m/s; 95% CI 0.005–1.131, p = 0.04) (Table 2, Fig. 1A, Supplementary Fig. 1). Furthermore, compared to baseline, Tcig smoking caused an increase in brachial systolic blood pressure and heart rate (p = 0.03 and p = 0.02, respectively) while HNBC puffing showed no significant changes on the above indices (p = 0.14 and p = 0.77) (Supplementary Fig. 2).

**Table 2:** Comparative acute effects of heat-not-burn cigarettes versus tobacco cigarette on arterial stiffness, oxidative stress, platelet activation, and exposure to CO in the acute study (n = 50).

	Baseline	Sham	HNBC	TCig
PWV (m/s)	9.7 ± 1.3	9.9 ± 1.7	10.2 ± 1.7^†^	10.8 ± 2.4*^†^
cSBP (mmHg)	121 ± 16	120 ± 15	119 ± 17.6	120 ± 17
Heart rate (bpm)	66 ± 9	66 ± 9	66 ± 8	70 ± 10*^†^
SBP (mmHg)	125 ± 15	125 ± 16	128 ± 19	130 ± 19*^†^
DBP (mmHg)	79 ± 10	78 ± 10	80 ± 11	82 ± 10
MDA (nmol/l)	1.34 ± 0.72	1.35 ± 0.83	1.28 ± 0.95	2.56 ± 0.85*^†^
PC (nmol/mg protein)	15.7 ± 5.8	14.5 ± 4.9	12.8 ± 5.2	13.9 ± 5.6
TxB2 (ng/ml)	378 ± 103	375 ± 105	362 ± 113	398 ± 105*^†^
CO (ppm)	14.9 ± 7.4	14.4 ± 3.8	14.1 ± 7.3	17.5 ± 7.8*^†^

*p < 0.05 of the overall model by ANOVA for the within subject effects of the tobacco products.

^†^p < 0.05 for the comparison of variables between baseline and after use of tobacco products by post hoc analysis using Bonferroni correction.

*PWV* carotid-femoral pulse wave velocity, *CO* carbon monoxide, *HNBC* heat-not-burn cigarette, *MDA* malondialdehyde, *TCig* tobacco cigarette, *TxB2* thromboxane B2, values are mean±SD.

#### Oxidative stress and platelet activation

In the acute setting, Tcig caused a significant MDA increase, in contrast with the lack of increase of MDA levels after HNBC puffing (baseline: 1.34 ± 0.72 vs Tcig: 2.56 ± 0.85, p = 0.03, vs HNBC: 1.28 ± 0.95 nmol/L, p = 0.55) (Table 2, Fig. 1B, Supplementary Fig. 3). Thus, MDA levels were found lower after HBNC than after Tcig (p = 0.02). Additionally, the MDA increase caused by Tcig was significantly correlated with the respective increase in PWV (r = 0.825, p < 0.001).

PC levels were unchanged during the acute phase of the study (p for the overall model, p = 0.54, Table 2).

Compared to baseline, acute Tcig smoking significantly increased TxB2 concentration; TxB2 levels did not change significantly following HNBC puffing (baseline: 378 ± 103 vs Tcig: 398 ± 105, p = 0.02, vs HNBC: 362 ± 113 pg/ml, p = 0.16). Thus, TXB2 levels were found lower after HBNC than after Tcig (p = 0.005) (Table 2, Fig. 1C, Supplementary Fig. 4).

#### CO exposure

In the acute phase, compared to baseline, exhaled CO levels were significantly elevated after Tcig smoking but were not affected by HNBC puffing (CO base: 14.9 ± 7.4 vs CO Tcig: 17.5 ± 7.8, p < 0.001, vs CO HNBC: 14.2 ± 7.3 ppm, p = 0.1) Thus, CO levels were found lower after HBNC than after Tcig (p < 0.001) (Table 2).

### Chronic study

#### Vascular function

There was a significant interaction between changes of FMD, CFR, TAC, central SBP and smoking status (HNBC or Tcig) at 1 month (F = 5.6, p = 0.01; F = 7.1, p = 0.001; F = 6.8, p = 0.001, and F = 5.4, p = 0.03, respectively).

**Figure 2:**
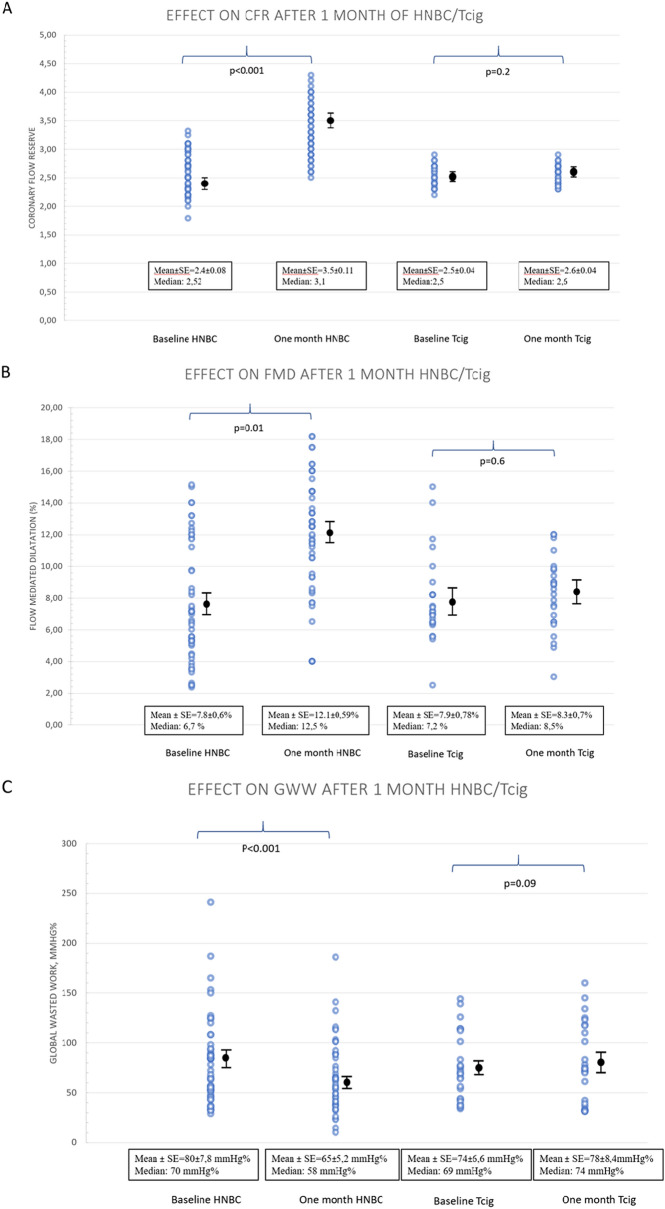
Progression of coronary flow reserve, flow mediated dilation, and myocardial work within 1 month of follow-up. Replacement of Tcig smoking with HNBC puffing for 1-month results in coronary and peripheral endothelial function improvement, along with wasted myocardial work reduction. *CFR* coronary flow reserve, *FMD* flow-mediated dilation, *GWW* global wasted work, *SE* Standard Error.

**Figure 3:**
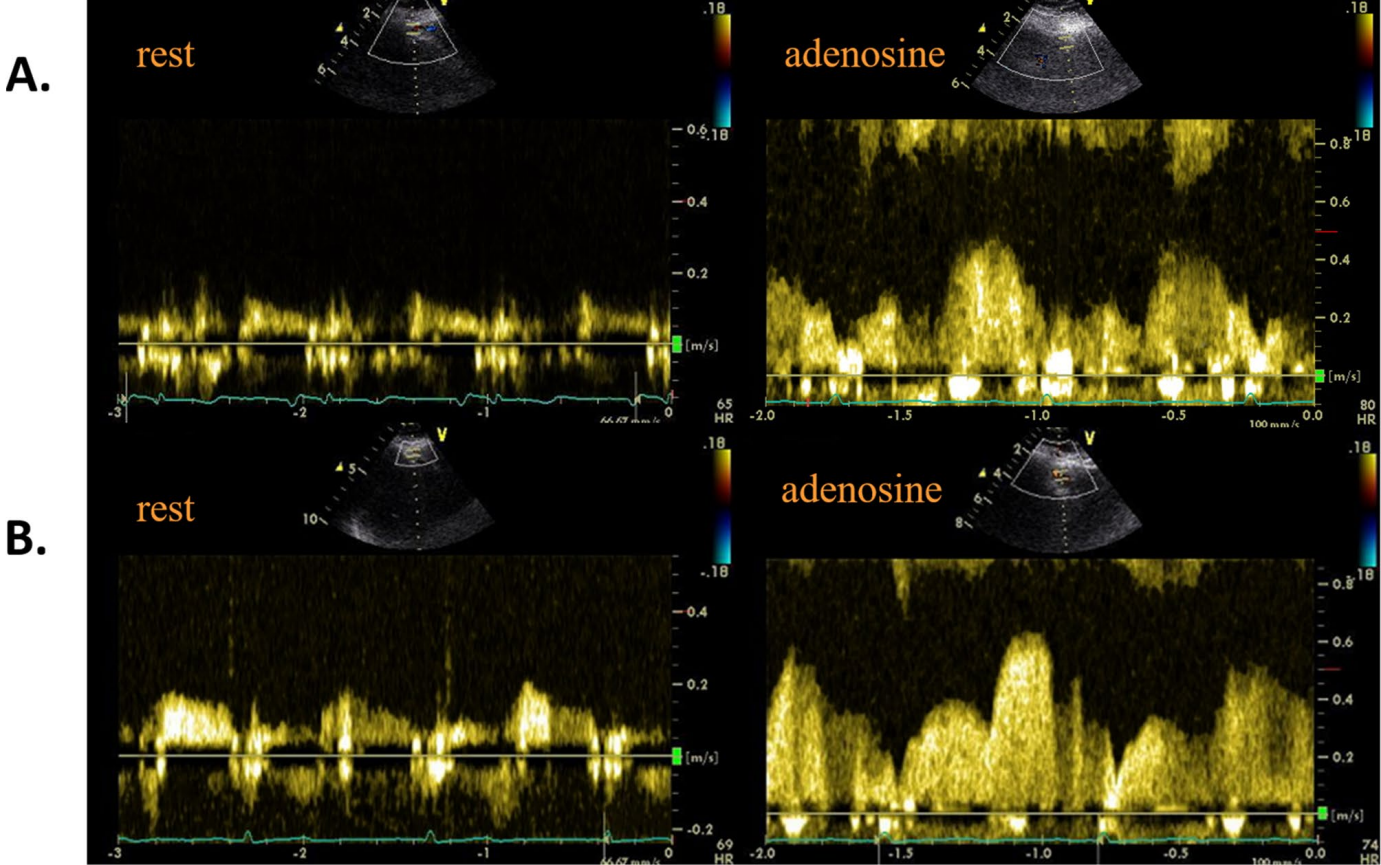
Coronary flow of left anterior descending artery at rest (left panel) and after adenosine infusion (right panel) for calculation of coronary flow reserve. Coronary flow of left anterior descending artery at rest (left panel) and after adenosine infusion (right panel) for calculation of coronary flow reserve at baseline (**A**) and after switching to HNBC for one month (**B**). The coronary flow reserve increased from 2.5 to 3.1 after 1 month of switching from tobacco cigarette to HNBC.

FMD and CFR were both significantly elevated within 1 month of switching to HNBC compared to tobacco smokers (difference in FMD = 4.3%; 95% CI 1.23–7.51, p = 0.009; difference in CFR = 0.98; 95% CI 0.23–1.80, p = 0.02) (Table 3, Figs. 2A,B and 3, Supplementary Figs. 5, 6).

**Table 3:** Arterial elasticity, myocardial deformation, endothelial function, and ventricular-arterial coupling progression after 1 month of switching from tobacco cigarette smoking to heat-not-burn product puffing (n = 50) compared to tobacco cigarette smokers (n = 25).

	Group	Baseline	One month	p-value
Body weight (kg)	HNBC	76.9 ± 2.4	77.1 ± 1.9	0.7
	Tcig	80.9 ± 3.4	80.5 ± 3.5	0.8
PWV (m/s)	HNBC	9.7 ± 1.3	10.1 ± 1.5	0.3
	Tcig	9.7 ± 1.6	10.2 ± 2.3	0.4
Central SBP (mmHg)	HNBC	121 ± 16	112 ± 17*	0.02
	Tcig	121 ± 16	123 ± 15	0.3
TAC (ml/mmHg)	HNBC	19.1 ± 4.2	20.9 ± 5.2*	0.03
	Tcig	17.6 ± 8.4	17.5 ± 7.6	0.2
Heart rate (bpm)	HNBC	66 ± 9	65 ± 8	0.4
	Tcig	67 ± 8	68 ± 8	0.8
SBP (mmHg)	HNBC	125 ± 15	126 ± 16	0.9
	Tcig	121 ± 13	122 ± 13	0.9
DBP (mmHg)	HNBC	79 ± 10	78 ± 9	0.7
	Tcig	76 ± 10	76 ± 10	0.9
GLS (%)	HNBC	− 19.9 ± 2.3	− 20.9 ± 2.5*	0.002
	Tcig	− 19.7 ± 1.3	− 20 ± 0.7	0.3
FMD (%)	HNBC	7.8 ± 4.3	12.1 ± 4.2*	0.01
	Tcig	7.9 ± 3.9	8.3 ± 3.5	0.6
CFR	HNBC	2.4 ± 0.6	3.5 ± 0.8*	< 0.001
	Tcig	2.5 ± 0.2	2.6 ± 0.2	0.2
PWV/GLS	HNBC	− 0.5 ± 0.1	− 0.48 ± 0.08*	< 0.001
	Tcig	− 0.49 ± 0.1	− 0.51 ± 0.1	0.4
GWI (mmHg%)	HNBC	1949 ± 315	1828 ± 320*	0.01
	Tcig	1954 ± 290	1986 ± 290	0.1
GCW (mmHg%)	HNBC	2214 ± 339	2156 ± 385	0.1
	Tcig	2202 ± 379	2180 ± 356	0.3
GWW (mmHg%)	HNBC	80 ± 55	65 ± 37*	< 0.001
	Tcig	74 ± 33	78 ± 42	0.09
GWE (%)	HNBC	95.8 ± 2.3	96.4 ± 1.8	0.1
	Tcig	95.5 ± 2	95 ± 2	0.5

*p < 0.05 for interaction with group, derived from post hoc analysis by ANOVA.

*HNBC* heat-not-burn cigarette, *TCig* tobacco cigarette, *CFR* coronary flow reserve, *PWV* carotid-femoral pulse wave velocity, *FMD* flow-mediated dilation, *GCW* constructive myocardial work, *GLS* global longitudinal strain, *GWE* myocardial work efficiency, *GWI* global myocardial work index, *GWW* wasted myocardial work, values are mean±SD.

Central SBP was significantly reduced in the HNBC group than in tobacco smoking group (difference = 10.4 mmHg-1; 95% CI 3.05–17.88, p = 0.02). TAC was significantly increased in the HNBC group than in tobacco smoking group (difference = 1.8 mL/mmHg; 95% CI 0.3–3.5, p = 0.04). Compared to baseline, PWV, HR, brachial SBP and DBP values were not reduced after 1 month of using HNBC (p > 0.05, Table 3, Supplementary Fig. 7). Compared to baseline, no significant changes were observed in all vascular markers within 1 month in the control group of tobacco smokers (p > 0.05, Table 3).

#### Myocardial function

There was a significant interaction between changes of GLS, PWV/GLS, GWI, GWW, GWE, and smoking status at 1 month (HNBC or Tcig) (p < 0.05 for all comparisons).

GLS was improved in the HNBC compared to the control group at follow-up (difference = 2.35%; 95% CI 0.23–4.48, p = 0.03) (Table 3, Supplementary Fig. 8).

PWV/GLS ratio, as a marker of ventricular-arterial interaction, was found to be improved within 1 month of switching to HNBC compared to conventional tobacco smoking (p = 0.03) (Table 3).

Moreover, GWI and GWW were both reduced within one month of HNBC use compared to conventional tobacco use (difference in GWI = 152 mmHg%; 95% CI 81.74–224.05, p = 0.001; difference in GWW = 19.72 mmHg%; 95% CI 4.35–35.08 p = 0.014) (Table 3, Fig. 2C). The increase in TAC was significantly correlated with the decrease in GWI (r = 0.344, p = 0.03). Compared to baseline, all myocardial deformation indices did not change significantly in the control group of tobacco smokers at 1 month (p > 0.05).

#### Oxidative stress and platelet activation

There was a significant interaction between changes of MDA, PC, TXB2 and smoking status (HNBC or Tcig) at 1 month (F = 7, p = 0.01; F = 4.8, p = 0.04; and F = 6.8, p = 0.01 respectively).

MDA and PC concentration significantly decreased in subjects switching to HNBC compared to tobacco smokers (difference MDA = 0.38 nmol/l; 95% CI 0.10–0.66, p = 0.009; PC = 7.73 nmol/mg protein; 95% CI 0.19–15.28, p = 0.04) (Table 4, Supplementary Fig. 9). Additionally, replacement of Tcig by HNBC caused a TxB2 reduction than tobacco smoking (difference = 45 pg/ml; 95% CI 5.28–86.31, p = 0.03) (Table 2, Supplementary Fig. 10). None of the aforementioned parameters changed in the control group at 1 month compared to baseline (p > 0.05 Table 4).

**Table 4:** Progression of exposure to CO, oxidative stress burden, and platelet activation after 1 month of switching from tobacco cigarette smoking to heat-not-burn product puffing.

	Group	Baseline	Follow-up	p-value
CO (ppm)	HNBC	14.9 ± 7.4	6.7 ± 6.4*	< 0.001
	Tcig	15.8 ± 4.9	17.4 ± 4.8	0.3
MDA (nmol/l)	HNBC	1.34 ± 0.72	1.11 ± 0.95*	0.01
	Control	1.43 ± 0.9	1.45 ± 0.83	0.3
PC (nmol/mg protein)	HNBC	15.7 ± 5.8	9 ± 4.4*	0.04
	Control	16.1 ± 8.8	17.2 ± 3.3	0.3
TxB2 (pg/ml)	HNBC	378 ± 103	323 ± 137*	0.01
	Control	417 ± 24	407 ± 16	0.4

*p < 0.05 for interaction with group, derived from post hoc analysis by ANOVA.

*CO* carbon monoxide, *MDA* malondialdehyde, *PC* protein carbonyls, *TxB2* thromboxane B2 values are mean ± SD.

At follow-up, the measured decrease in plasma MDA levels was significantly positively correlated with the increase in FMD (r = 0.51, p = 0.03).

#### CO exposure

There was a significant interaction between changes of CO and smoking status (HNBC or Tcig) at 1 month (F = 8.5, p = 0.001). Compared to baseline, CO measurements were reduced after switching to HNBC (p < 0.001), while remaining unchanged in controls subjects (p = 0.2) (difference in CO between groups: 10.42 ppm; 95% CI 3.07–17.76, p = 0.007) (Table 4).

## Discussion

In the acute crossover phase of this study, it was demonstrated that a single HNBC puffing resulted in a smaller increase of pulse wave velocity compared to tobacco cigarette smoking and was not associated with further impairment of oxidative stress and platelet activation or increased exposure to CO, compared to baseline. Conversely, acute smoking of a tobacco cigarette had detrimental effects on the examined markers of vascular function, oxidative stress, and platelet activation. Furthermore, in the chronic phase of this study, switching from Tcig to HNBC for 1 month was associated with improvement in endothelial function, coronary flow reserve, arterial compliance, and myocardial work, as well as with reduction of oxidative stress burden, platelet activation, and exposure to CO. These changes were not evident in the parallel control group of subjects who continued smoking tobacco cigarettes for a month. Interestingly, the reduction in oxidative stress burden after switching to HNBC was associated with the respective improvement of endothelial function, while the increase in arterial compliance was associated with the concomitant reduction of myocardial work.

Arterial elasticity is impaired in the context of both acute and chronic smoking, as assessed by carotid to femoral PWV ^2,34^. The use of electronic cigarettes has shown less detrimental effects on arterial elasticity compared to smoking tobacco cigarettes ^2^. Increased arterial stiffness has been shown to be reversible, albeit after a considerable period of abstinence from smoking, spanning from 2 to 10 years ^2,34–37^. To our knowledge, this is the first study to demonstrate that acute HNBC puffing is associated with a smaller increase of PWV compared to tobacco smoking. HNBC puffing increases nicotine blood levels. Nicotine activates the sympathetic nervous system by acting via splanchnic nerves to the adrenal medulla and stimulates the release of epinephrine ^38^. Acute elevation of nicotine levels has been linked to endothelial dysfunction ^39^ leading to reduced nitric oxide release and thus contributes in addition to increased sympathetic activity to increased vascular tone. These mechanisms likely underlie the increase of PWV after acute HNBC puffing. Conversely, the deleterious acute effects of smoking a tobacco cigarette on arterial stiffness were in line with the respective oxidative stress burden accentuation, underlining their interplay in our acute study ^2,40^. TAC constitutes an index of arterial compliance of the entire arterial tree and entails physiologic and prognostic significance ^9^. To our knowledge, this is the first study to highlight that replacement of Tcig with a HNBC results in improved TAC and central SBP, suggesting a less detrimental effect of this novel smoking product on left ventricular afterload. The discrepancy between lack of improvement of PWV and increase of TAC in our study may be explained by the different vascular beds these indices represent. On the one hand, PWV assesses the aortoiliac segment of the arterial tree ^41^; on the other hand, TAC incorporates the effects of the large, central, elastic arteries as well as the small, peripheral, muscular arteries ^9^. Thus, improved arterial compliance of the peripheral arterial system could improve TAC, without affecting PWV.

Augmentation of oxidative stress is a key pathophysiological mediator of cardiovascular harm caused by acute and chronic smoking ^2,34^. MDA is the product of cellular membrane phospholipids peroxidation and thus a valid and reproducible marker of lipid peroxidation ^17^. PCs represent an irreversible form of protein modification that are formed early during oxidative stress conditions, are relatively stable in contrast to lipid peroxidation products, are not a result of one specific oxidant, and thus they are considered a sensitive and reproducible marker of total protein oxidation ^18^. In our previous studies, both MDA and PCs were increased in smokers and their levels were reduced either after nicotine replacement treatment or varenicline use in medically-assisted smoking cessation programs ^34^ or after switching to electronic cigarette smoking. ^2,42^. Thus, plasma concentrations of MDA ^17^ and PC ^18^ are valid markers of oxidative stress burden related to tobacco smoking. Recently, evidence has arisen that acute HNBC puffing is less detrimental to oxidative stress equilibrium compared with Tcig smoking, as assessed by Nox2-derived peptide, vitamin E, and HBA levels ^32^. Similarly, our study demonstrated that MDA levels were not elevated acutely after using an HNBC, in contrast with Tcig use. Furthermore, in our chronic study, we demonstrated reduced MDA and PC levels within 1 month of replacing Tcig with HNBC.

Increased platelet activation plays a significant role in promoting atherosclerosis and thrombotic complications, and is enhanced by Tcig smoking ^5^. A recent study suggests that HNBC invokes platelet activation to a lesser degree than Tcig, as evident by comparatively lesser increase in soluble CD40 ligand and soluble P-selectin, as markers of platelet activation ^32^. Plasma TxB2 is a valid marker of platelet activation ^19^. In agreement with the aforementioned study ^32^, in our acute study, we did not detect significant changes in plasma TxB2 acutely after HNBC puffing compared to baseline. Conversely, we observed the increasedTXB2 levels after Tcig smoking. Further expanding on this finding, plasma TxB2 levels were significantly reduced after 1 month of HNBC use while no changes were observed in the parallel group of tobacco smokers in the same period.

Exhaled CO levels constitute a widely used marker of acute smoking status ^43^. In addition, inhaled CO has been associated with endothelial damage and pathological capillary permeability ^34^. A recent study investigating the BoE footprint of HNBC highlighted that exhaled CO levels within 5 days of switching from Tcig to HNBC are equal to those measured after total abstinence from tobacco products ^24^. Similarly, in our study, acute HNBC use did not increase exhaled CO levels, while CO levels were significantly reduced at the 1-month follow-up examination, approaching though the levels previously reported for passive-smokers ^24,43^. This finding suggests that some of the HNBC users were substantially exposed to passive smoking in their family and/or working environment despite adherence to HNBC use during the chronic study.

Endothelial dysfunction is a prominent end-point of the pathophysiological cascades activated by Tcig smoking ^4^. In this regard, FMD and CFR evaluation reflecting endothelial and coronary microcirculatory function respectively, possess unequivocal diagnostic and prognostic value ^10,12^. Early investigation of the interaction between HNBC and endothelial function has not produced consistent results. On the one hand, acute HNBC puffing has been shown to impair FMD to a lesser extent than Tcig ^32^. On the other hand, an animal model study recognised HNBC and Tcig as equally detrimental on endothelial function ^33^. Our study found a significant increase in FMD after switching to HBNC use for 1 month, in accordance with recently published evidence of FMD improvement after replacing TCig smoking with electronic-cigarette vaping ^44^. Interestingly, FMD improvement was positively correlated with MDA reduction at 1 month, suggesting the contribution of reduced oxidative stress to improvement of endothelial function. Furthermore, the current study is the first to our knowledge that has demonstrated a significant improvement in CFR within 1 month of switching to HNBC. Studies strongly support the independent prognostic value of a reduced CFR for adverse cardiovascular outcome ^13^.

Novel markers of ventricular arterial-interaction incorporate measurements of afterload in the investigation of myocardial function using speckle tracking echocardiography; the shortcomings of ejection fraction assessment and the diagnostic pitfalls arising from the load-dependent nature of global longitudinal strain estimation are overcome. Thus, a non-invasive and more clinically feasible method of myocardial work estimation is provided by constructing longitudinal myocardial strain-pressure curves using speckle tracking imaging ^16^. In our study myocardial efficiency was improved after switching to HNBC for one month, as shown by the respective improvement of the GLS ^11^ and the reduction of the myocardial work index (GWI), global wasted myocardial work (GWW) and the increase of the global myocardial work efficiency (GWE) as assessed by the respective strain-pressure curves. Interestingly, improved GWI was associated with increasing TAC values Thus, the reduction of GWI after switching to HNBC may be attributed to the reduction of LV afterload as indicated by the concomitant reduction of central SBP and increase of TAC at one month. Furthermore, the observed improvement of the ratio PWV/GLS after one month of HNBC use also suggests an improvement in ventricular-arterial interaction ^11^. Thus, in our study we observed that the improvement of vascular function and oxidative stress, likely on the grounds of reduced CO exposure, evolved in parallel with the improvement of myocardial work efficiency after switching from the traditional tobacco smoking to HNBC for one month. Alternatively, a potential reduction in the blood nicotine levels after switching to HNBC from Tcig smoking may have also accounted for the improvement of cardiac and vascular markers in our chronic study.

In an elegant study by Frati et al. ^45^, the use of unsupervised machine learning techniques identified different clusters of individuals within a larger study cohort with a similar within each cluster but different among clusters responses to HNBC or e-cigarette smoking for various vascular and biochemical markers. The identification of clusters of subjects with favorable or unfavorable responses to novel smoking products for surrogate markers of cardiovascular function may facilitate the definition of the potential individual specific features of cardiovascular safety of these smoking products.

## Methods

### Design

Our study was an acute independent, randomised, cross-over trial followed by a chronic case control follow-up study.

According to our initial Study design, (ClinicalTrials.gov, NCT03452124) the primary outcome for the acute study was the effect of HNBC in comparison to Tcig on PWV, while primary outcome for the chronic study was the effect of use of HNBC for one month in comparison to Tcig smoking for the same period on LV deformation. The study was approved by the Attikon University Hospital scientific ethics committee (Approval number: 2874/06-12-17), funded by the Hellenic Society of Lipidology of Atherosclerosis and Vascular Disease. The study was conducted according to the Declaration of Helsinki and written informed consent was provided by the participants. Our study was registered on https://clinicaltrials.gov/, NCT03452124, 02/03/2018.

### Study population

**Figure 4:**
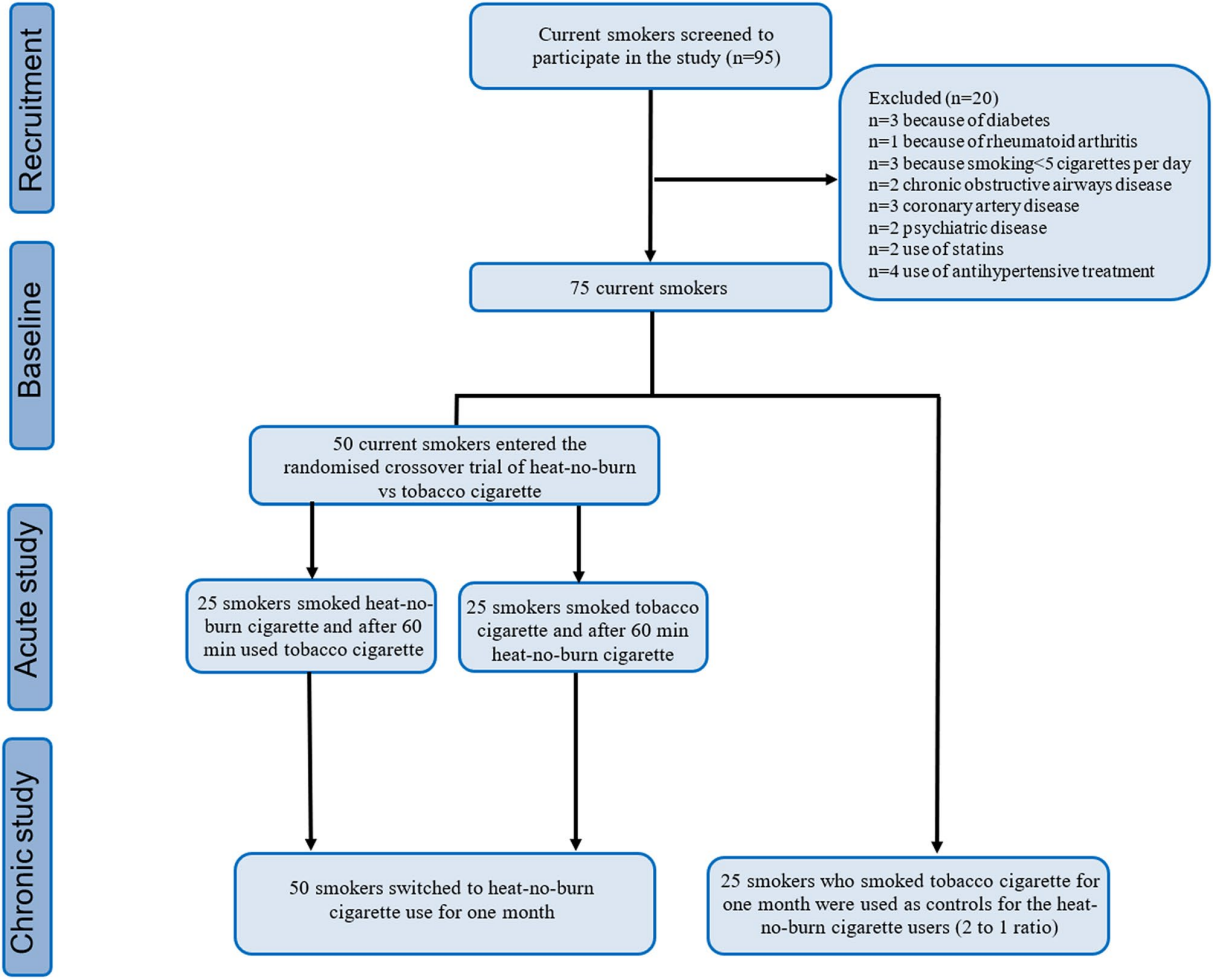
Flow chart of the study population. *HNBC* heat-not-burn cigarette, *TCig* tobacco cigarette.

Out of 95 screened smokers attending the Attikon University Hospital smoking cessation unit, 50 current smokers (age: 48 ± 5 years, 53% female, 27 ± 9 cigarettes/day, 29 ± 9 pack-years) and 25 controls smokers (age: 46 ± 14 years, 53% female, 26 ± 8 cigarettes/day, 30 ± 8 pack-years) with no intention to quit smoking were included in our study (Fig. 4). Smoking status was verified by way of self-reported smoking burden per day and exhaled carbon monoxide (eCO) concentration measurement [parts per million (ppm), Bedfont Scientific, Maidstone, Kent UK] (exclusion criteria: < 5 cigarettes per day end exhaled CO < 10 ppm). Exclusion criteria included history of cardiovascular disease, hepatic or renal failure, active neoplasia, alcohol abuse, psychiatric illness, pregnancy, breastfeeding, cigar smoking, or the presence of any additional risk factor for cardiovascular disease (dyslipidaemia: total cholesterol > 200 mg/dl or the use of cholesterol-lowering agents; hypertension: blood pressure > 140/90 mmHg or use of anti-hypertensive drugs; diabetes mellitus: fasting plasma glucose > 125 mg/dl or use of antidiabetic drugs).

### Study protocol

**Figure 5:**
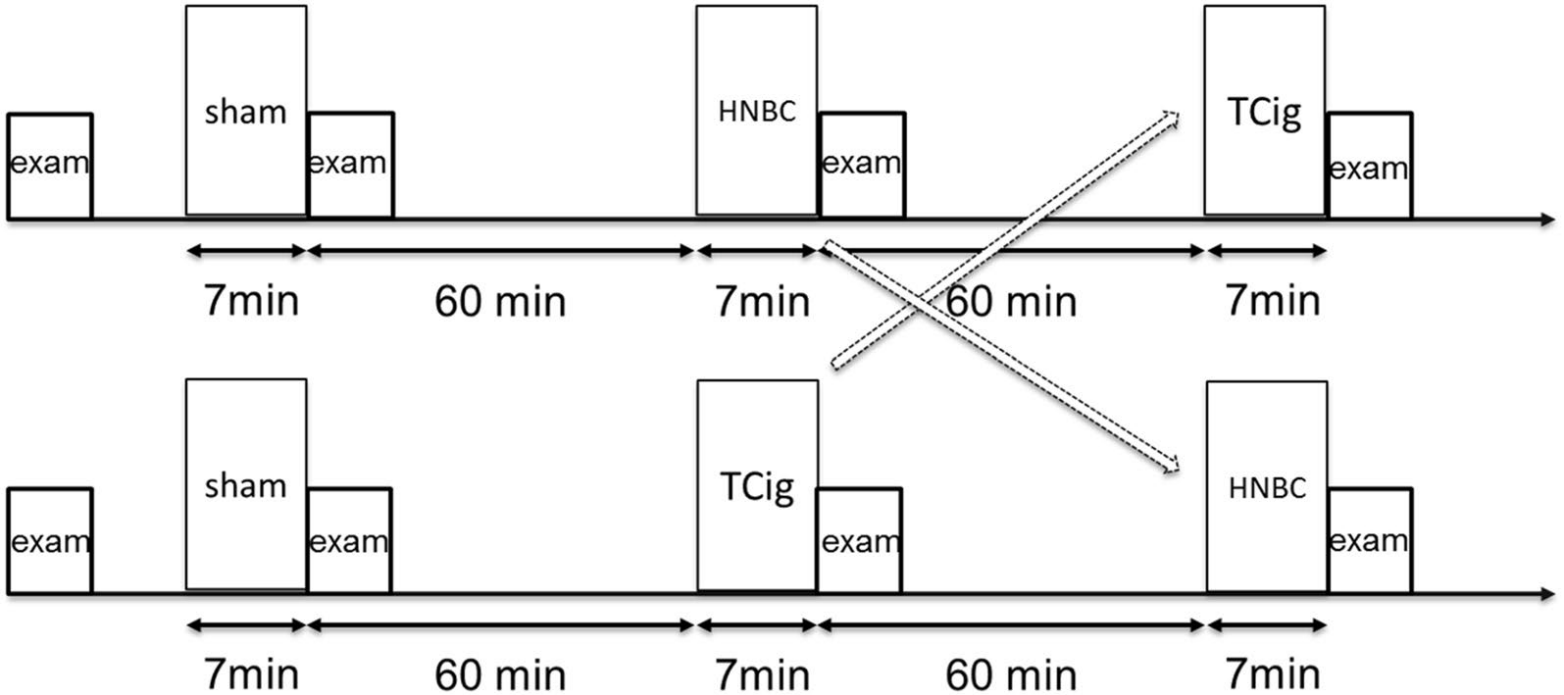
Acute phase protocol. Following an initial sham smoking session, subjects were randomised to either a heat-not-burn or traditional cigarette smoking session; following a washout period of 60 min, they were crossed-over to the alternative smoking session. Each session was followed by a vascular stiffness examination and blood-sampling for oxidative stress and platelet activation assessment. *Exam* examination, *HNBC* heat-not-burn cigarette, *TCig* tobacco cigarette.

Our study included an acute and a chronic phase. The acute phase of the study entailed an initial sham smoking session of inhaling on a non-lighted cigarette for 7 min, simulating the mean duration of a traditional cigarette smoking. In the acute phase, the participants were thereafter randomised into either a tobacco cigarette (Tcig) smoking session, using a single mainstream Tcig [Marlboro Red, Papastratos-Philip Morris International (PMI), Athens, Greece], or a single HEETS stick (PMI, amber flavour) puffing session (HNBC) using a commercially available HNBC (IQOS, PMI). Randomization was performed by an attending research nurse using a table of random numbers as reproduced from the online randomization software http://www.graphpad.com/quickcalcs/index.cfm, as previously published ^45^. Proper use of IQOS device was demonstrated by a qualified research nurse, who also served as a supervisor during the HNBC puffing session. After a washout period of 60 min, the subjects were crossed over to the alternative session (Tcig or HNBC, Fig. 5). Vascular studies and blood sampling were completed during 20-min at baseline before initiation of smoking and within the wash-out period. The decision about the washout period duration was based on evidence that the acute effects of a smoking on arterial function resolve within 60 min ^41^.

For the chronic phase, all participants of the acute phase, were instructed to replace Tcig smoking with HNBC puffing for 1 month and were compared with an external group of 25 Tcig smokers, with no intention to quit smoking, (2:1 ratio) before and after 1 month.

Exhaled CO, arterial elasticity and blood sampling for oxidative stress and platelet activation, which were assessed at baseline, after each smoking session of the acute phase and at the chronic phase. Endothelial function, coronary flow reserve and myocardial deformation were assessed at baseline and after 1 month, in the chronic study. Arterial elasticity, myocardial, endothelial function examinations were executed by a single, blinded-to-treatment and to values of measured biomarkers, operator. In the acute study the time elapsed between smoking (Tcig or HNBC) and assessment of vascular markers was approximately 10 min. For the chronic study, participants were instructed to abstain from smoking (Tcig or HNBC) in the morning before the vascular and echocardiography assessment. HNBC adherence was assessed by asking the participants to answer a questionnaire regarding the daily use of HNBC sticks as well as by measurement of the exhaled CO in each participant during a clinic visit at days 15 and 30 of the chronic study. The HNBC sticks were provided to the participants by the investigators at baseline and at day 15 during a clinic visit and after the participant had returned the empty boxes of the used HNBC sticks.

### Endothelial function

Brachial artery FMD was ultrasonically assessed in line with published methodology recommendations ^17^ and expressed as the percent increase of the baseline arterial diameter. Intra-observer variability of the brachial artery diameter was 0.1 ± 0.12 mm.

### Coronary flow reserve

CFR was measured by transthoracic Doppler echocardiography, analysing colour-guided pulse-wave Doppler signals derived from long axis apical projections. The maximal velocity end velocity–time integral in the distal left anterior descending artery were recorded at rest and following intravenous adenosine administration, according to published methodology ^46^ and quantified as the ratio of hyperaemic to resting maximal diastolic velocity. Coronary flow reserve assessment of left anterior descending artery was feasible in all subjects of the study, though in 3 out of 75 participants, we had to use intravenous contrast (Sonovue, Bracco, Italy) to improve Doppler imaging of the coronary flow.

### Arterial stiffness

Carotid-to-femoral PWV was estimated according to a previous published methodology (Complior, Alam Medical, Vincennes, France) ^40^, by computing the ratio of the distance between the carotid and femoral pulse palpation site to the pulse wave transit time (m/s). The Cointra-observer variability was 6%, in accordance with existing evidence in similar studies ^34^. Total arterial compliance (TAC) was evaluated utilising the stroke volume (SV) to pulse pressure (PP) ratio (SV/PP), based on the two-element Windkessel model ^9^; SV measurements were derived from two-dimensional echocardiography.

In the acute and chronic study assessment of vascular markers (FMD, PWV and PP) was performed in a random order.

### Myocardial deformation

Myocardial deformation was assessed by way of 2-dimensional strain measurement, with speckle-tracking analysis by dedicated software (Echopac 203, GE Horten Norway). LV apical 2-, 3-, and 4-chamber views at ≥ 50 frames per second frame rate were acquired and the global longitudinal strain (GLS) was calculated from the respective apical views, applying previously published methodology ^17,34^. PWV/GLS ratio (− m/s%) was calculated as a marker of ventricular arterial interaction ^36^.

### Myocardial work

Myocardial work (MW) was estimated by combining echo-derived left ventricular (LV) strain with brachial blood pressure to construct LV strain-pressure curves non-invasively, following recently published methodology ^16^. Brachial cuff systolic pressure measurements provide the peak systolic LV pressure value, which is combined with the input of valvular timing events that define isovolumetric and ejection phases, allowing the construction of an LV pressure curve. This is combined with LV strain data into a pressure-strain loop (PSL), the area within which represents MW. Global MW index (GWI) is defined as the work within the LV PSL from mitral valve closure to mitral valve opening, while constructive MW (GCW) is the component of MW that contributes to LV ejection. On the contrary, wasted MW (GWW) is the work wasted as myocyte lengthening during systole, which does not contribute to LV ejection. MW efficiency (GWE) is defined as the ratio of GCW to the sum of GCW and GWW [GWE = GCW/(GCW + GWW)].

### Oxidative stress

A commercially available spectrophotometry kit (Oxford Biomedical Research, Rochester Hills, Mich, colorimetric assay for lipid peroxidation; measurement range 1–20 nmol/l) was used to determine plasma MDA levels. Plasma PC levels were measured by spectrophotometrical assessment of 2,4-dinitrophenylhydrazine PC derivatives, as previously published ^40^, and results were expressed as nmol/mg protein. For MDA and PC, the intra-assay variability was 3.39% and 4.52%, respectively and the inter-assay variability was 4.75% and 5.93%, respectively.

In the chronic study two-dimensional echocardiography preceded the coronary flow reserve assessment, after completion of vascular studies.

### Platelet activation

A commercially available ELISA kit was used to measure blood levels of Thromboxane B2 (Thromboxane B2 EIA Kit Cayman Ann Arbor MI USA) with an assay range 1.6–1000 pg/ml.

### Statistical analysis

We used the software STATA v.11 and SPSS v.22 in order to procced to data analysis. We used Shapiro–Wilk test to examine if values had normal distribution, and Levene test for evaluating data homoscedasticity, as it was previously published ^34^. Non-parametric variables were transformed into rank for analysis. Two tailed tests with p < 0.05 were used during data acquisition. ANOVA (general linear model, SPSS 22, SPSS Inc, Chicago, Ill) for repeated measurements was applied for analysis of the examined indices of vascular, myocardial, and endothelial function, as well as biochemical markers (at baseline, after each smoking session, and at the 1-month follow-up examination) with the parameter of time used as a within-subject factor. The Greenhouse–Geisser correction was used when the sphericity assumption, as assessed by Mauchly’s test, was not met. We used parametric (Pearson r) and non-parametric (Spearman rho) correlation coefficients to examine cross-sectional associations. Multiple comparisons of baseline values of study markers to values after sham, Tcig or HNBC in the acute study were performed with Bonferroni correction. A p-value of < 0.05 was considered statistically significant. The ordering of intervention (begin with HBNC versus begin with Tcig) and the brachial systolic blood pressure (SBP) were included as covariates in the model and the respective p-value for their interaction with the overall model was calculated. T-test was performed to compare the absolute or percent changes of the parameters evaluated in the chronic phase of the study. Inter- and intra-observer variabilities (%) of vascular, myocardial function, and biochemical markers were calculated as the SD of the differences between the first and second measurements, and expressed as a percentage of the average value in 30 healthy volunteers.

### Power analysis

For the acute study, we planned a study of a continuous response variable from matched pairs of study subjects (HNBC and Tcig users). In a pilot study of ten smokers randomised to HNBC or Tcig and then crossed-over to the alternate smoking (Tcig or HNBC) the difference in the PWV of matched pairs after HNBC and Tcig smoking was normally distributed with standard deviation 1.3 and the true difference in the mean PWV of the matched pairs after HBNC and Tcig smoking was 0.54 m/s. Thus, we needed to study 47 pairs of subjects to be able to reject the null hypothesis that this response difference in PWV after HNBC and Tcig is zero with probability (power) 0.8. The Type I error probability associated with this test of this null hypothesis is 0.05.

For the chronic study, we planned a study of a continuous response variable from independent control (Tcig smokers) and experimental subjects (HNBC users) with 0.5 control(s) per experimental subject. In a pilot study of 10 HNBC and 5 Tcig smokers the response for the GLS within each subject group was normally distributed with a standard deviation of 3.1% and the true difference of GLS means between the HNBC and Tcig smokers was 2.3%. Thus, we needed to study at least 50 experimental and 25 control subjects to be able to reject the null hypothesis that the population means of the HNBC and Tcig smoking groups are equal with a probability (power) of 0.8. The Type I error probability associated with this test for this null hypothesis is 0.05, as previously published ^34^.

### Ethical approval

It was approved by the Attikon University Hospital scientific ethics committee (Approval number: 2874/06-12-17), registered on https://clinicaltrials.gov/ (NCT03452124, 25/02/2018). The study was conducted according to the Declaration of Helsinki and written informed consent was provided by the participants.

## Study limitations

Our study was a single centre study. Its design does not permit to explore definite causative associations among changes in oxidative stress, vascular and myocardial function markers. We should also acknowledge as a study limitation the lack of measurement of cotinine blood or urine levels to appraise for the actual number of products smoked by our study participants and to clarify whether differences in circulating nicotine levels in addition to differences in CO production after HNBC and Tcig smoking mediate the observed differences of cardiovascular markers in our study. Long term follow-up is needed to assess whether the observed improvement in vascular and myocardial function, after switching to HBNC, is associated with reduced cardiovascular events. Cluster analysis using unsupervised machine learning techniques to identify subgroups of participants with favourable or unfavourable responses to HNBC use for the measured vascular, cardiac and biochemical markers was not performed.

## Conclusion

Acute HNBC puffing showed a less detriment effect on arterial elasticity compared to Tcig and did not cause a further augmentation of oxidative stress burden, platelet activation, and exposure to CO compared to baseline, in contrast to acute smoking of tobacco cigarette. Switching from Tcig to HNBC for one month resulted in improved endothelial function, oxidative stress burden as well as in reduction of platelet activity and exposure to CO, while caused an improvement in coronary flow reserve and myocardial work efficiency compared with tobacco smoking.

## Supplementary Information

Supplementary Information.

## Data Availability

The datasets generated during and/or analysed during the current study are available from the corresponding author on reasonable request. Anonymised data are only available upon request from the authors.
