# Supplementary material for: Differential effects of heat-not-burn and conventional cigarettes on coronary flow, myocardial and vascular function
Source: Sci Rep. 2021 Jun 3;11:11808. doi: 10.1038/s41598-021-91245-9 (PMC8175445; doi:10.1038/s41598-021-91245-9)
Supplement: Supplementary file 1 — Supplementary Information. [file 41598_2021_91245_MOESM1_ESM.docx]

**Differential effects of heat-not-burn and conventional cigarettes on coronary flow, myocardial and vascular function.**

**Ignatios Ikonomidis^1^, Dimitrios Vlastos^2^, Gavriela Kostelli^1^, Kallirhoe Kourea^1^, Konstantinos Katogiannis^1^, Maria Tsoumani^3^, John Parissis^1^, Ioanna Andreadou^3^, Dimitrios Alexopoulos^1^**

**From:**

1. **2nd Cardiology Department, Attikon Hospital, National and Kapodistrian University of Athens, Medical School, Greece**
2. **Department of Cardiac Surgery, Royal Brompton Hospital, Imperial College, London, UK**
3. **Department of Pharmaceutical Chemistry, National and Kapodistrian University of Athens, School of Pharmacy, Athens, Greece.**

**Brief title: Differential effects of heat-not-burn and conventional cigarettes.**

**Word count:** 5082

**Address for correspondence**

Dr Ignatios Ikonomidis, MD, FESC

2nd Cardiology Department, Attikon Hospital, University of Athens,

Rimini 1, Haidari, 12462 Athens, Greece

tel: +30210 5832187. fax: +30210 5832192

Email address: [ignoik@gmail.com](mailto:ignoik@gmail.com)


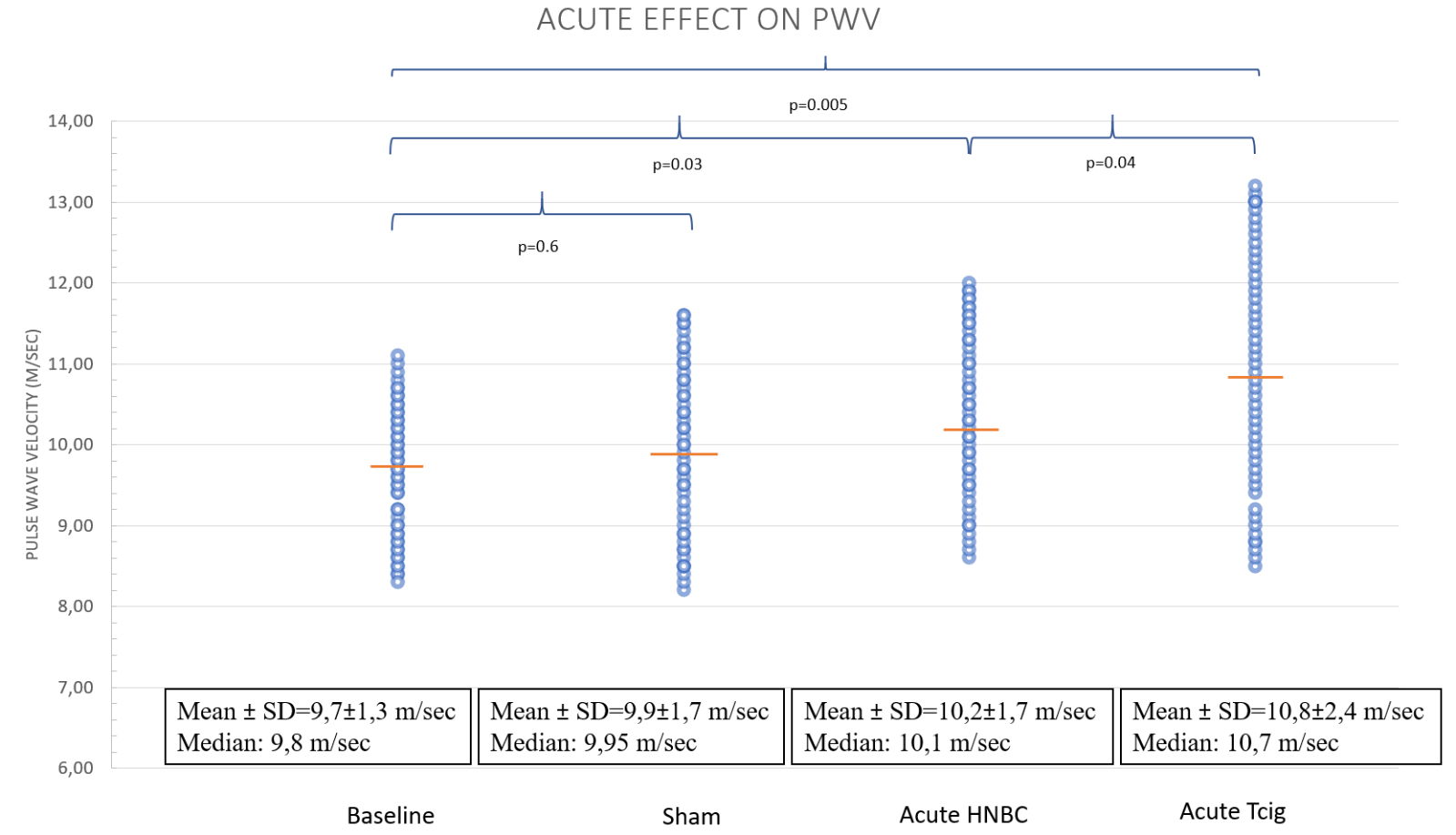


SUPPLEMETARY FIGURE 1. Scatterplot that represents PWV values (m/sec) of the study population during the acute study, at baseline, after sham smoking, after HNBC puffing and after Tcig smoking. Red line represents the mean value. In the box we report the mean ±SD and the median.

In the acute phase, the participants were randomised into either a Tcig smoking session or a single HNBC puffing session. After a washout period of 60 minutes, the subjects were crossed over to the alternative session (Tcig or HNBC). Vascular studies and blood sampling were completed during 20-minutes at baseline before initiation of smoking and within the wash-out period. PWV = carotid-femoral pulse wave velocity; HNBC = heat-not-burn cigarette; Tcig = tobacco cigarette; SD = Standard Deviation.


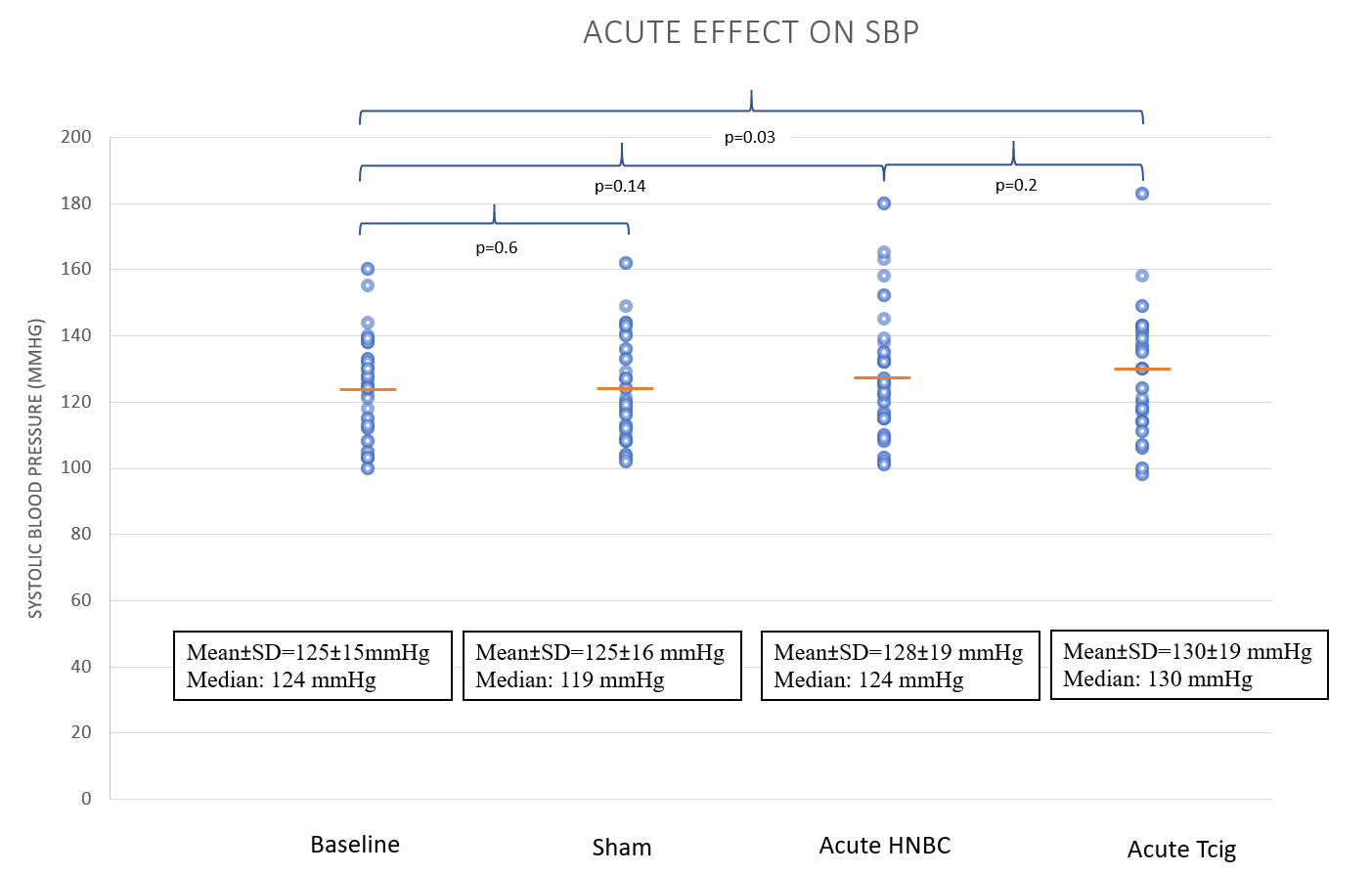


SUPPLEMETARY FIGURE 2. Scatterplot that represents SBP values (mmHg) of the study population during the acute study, at baseline, after sham smoking, after HNBC puffing and after Tcig smoking. Red line represents the mean value. In the box we report the mean ±SD and the median.

In the acute phase, the participants were randomised into either a Tcig smoking session or a single HNBC puffing session. After a washout period of 60 minutes, the subjects were crossed over to the alternative session (Tcig or HNBC). Vascular studies were completed during 20-minutes at baseline before initiation of smoking and within the wash-out period. SBP = Systolic Blood Pressure; HNBC = heat-not-burn cigarette; Tcig = tobacco cigarette; SD = Standard Deviation.


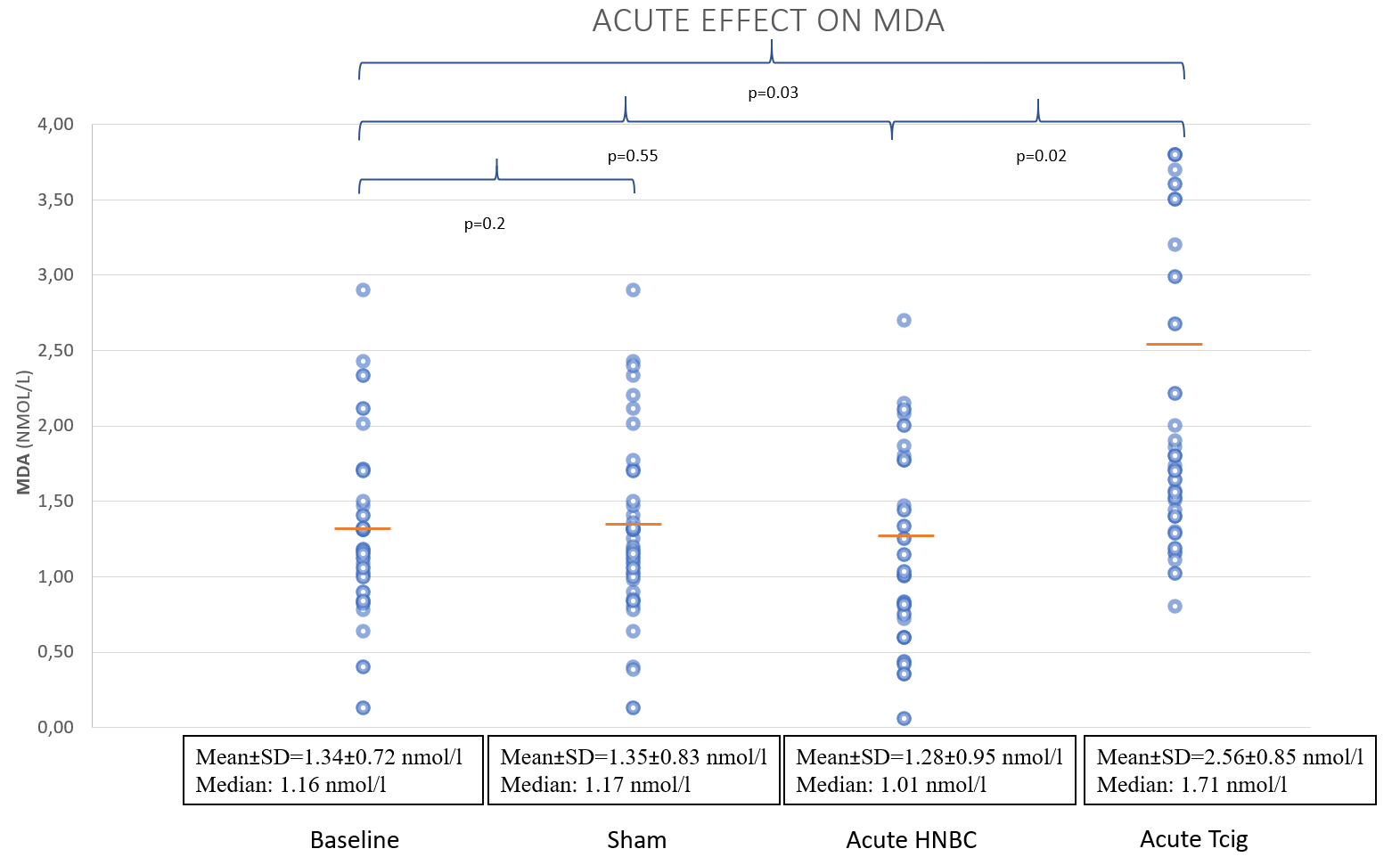


SUPPLEMETARY FIGURE 3. Scatterplot that represents the MDA values (nmol/L) of the study population during the acute study, at baseline, after sham smoking, after HNBC puffing and after Tcig smoking. Red line represents the mean value. In the box we report the mean ±SD and the median.

In the acute phase, the participants were randomised into either a Tcig smoking session or a single HNBC puffing session. After a washout period of 60 minutes, the subjects were crossed over to the alternative session (Tcig or HNBC). Blood sampling were completed during 20-minutes at baseline before initiation of smoking and within the wash-out period. MDA = malondialdehyde; HNBC = heat-not-burn cigarette; Tcig = tobacco cigarette; SD = Standard Deviation.


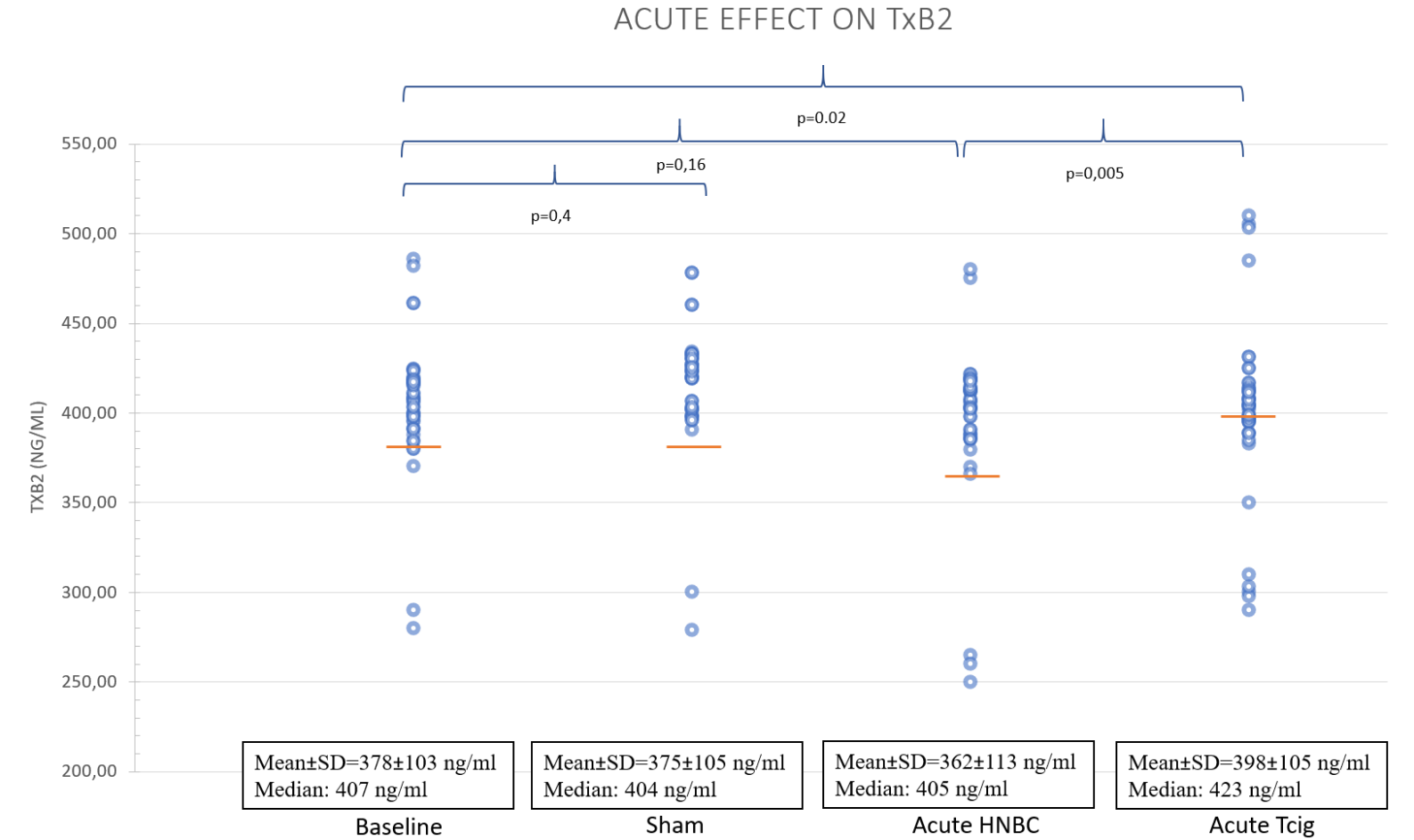


SUPPLEMETARY FIGURE 4. Scatterplot that represents the TxB2 values (ng/mL) of the study population during the acute study, at baseline, after sham smoking, after HNBC puffing and after Tcig smoking. Red line represents the mean value. In the box we report the mean ±SD and the median.

In the acute phase, the participants were randomised into either a Tcig smoking session or a single HNBC puffing session. After a washout period of 60 minutes, the subjects were crossed over to the alternative session (Tcig or HNBC). Vascular studies and blood sampling were completed during 20-minutes at baseline before initiation of smoking and within the wash-out period. TxB2 = thromboxane B2; HNBC = heat-not-burn cigarette; Tcig = tobacco cigarette; SD = Standard Deviation.


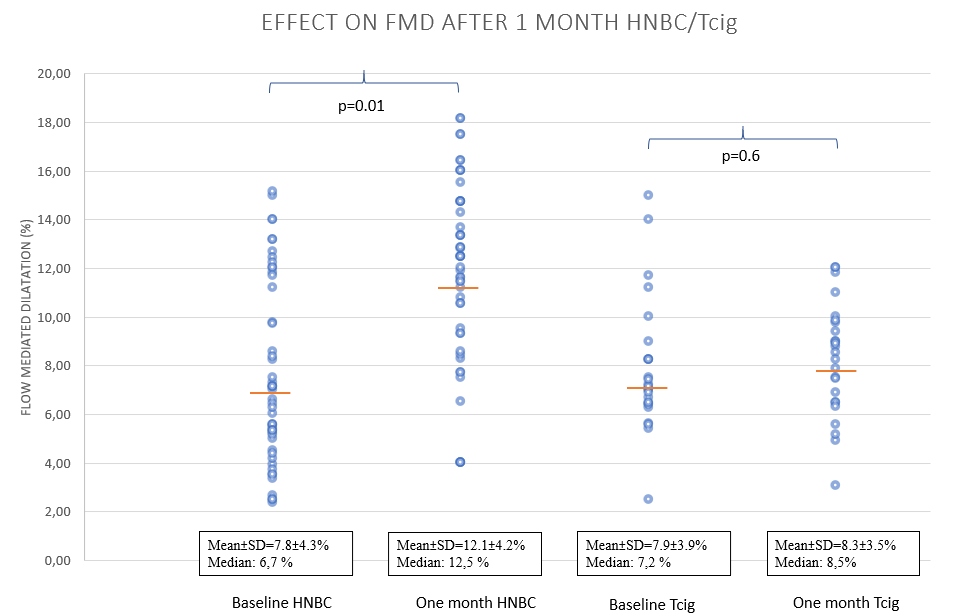


SUPPLEMETARY FIGURE 5. Scatterplot that represents the FMD values (%) of the study group and the control group during the chronic study. For the chronic phase, all participants of the acute phase (50 subjects), were instructed to replace Tcig smoking with HNBC puffing for 1 month and were compared with an external group of 25 Tcig smokers, before and after one month. Red line represents the mean value. In the box we report the mean ±SD and the median. FMD = Flow Mediated Dilatation; HNBC = heat-not-burn cigarette; Tcig = tobacco cigarette; SD = Standard Deviation.


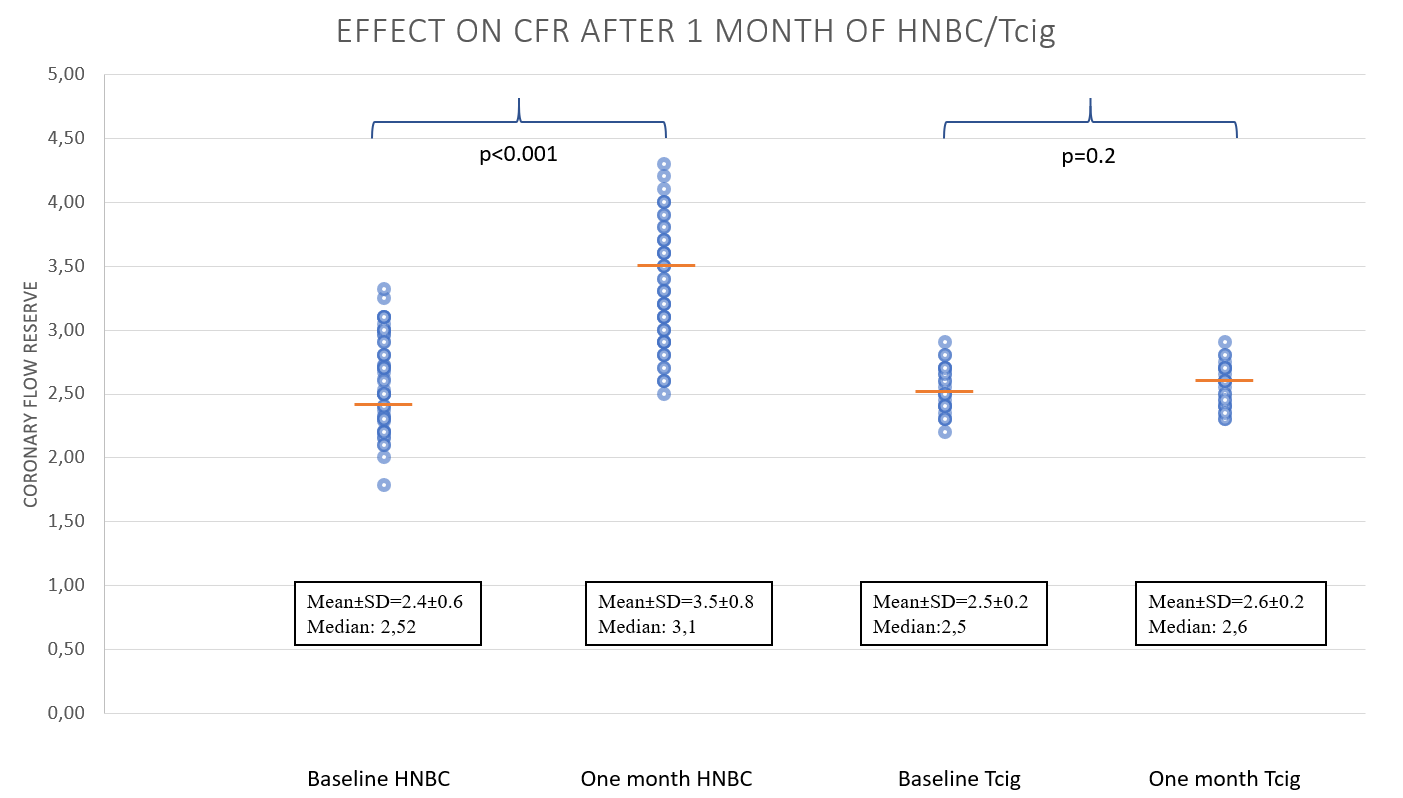


SUPPLEMETARY FIGURE 6. Scatterplot that represents CFR values of the study group and the control group during the chronic study. For the chronic phase, all participants of the acute phase (50 subjects), were instructed to replace Tcig smoking with HNBC puffing for 1 month and were compared with an external group of 25 Tcig smokers, before and after one month. Red line represents the mean value. In the box we report the mean ±SD and the median. CFR = Coronary Flow Reserve; HNBC = heat-not-burn cigarette; Tcig = tobacco cigarette; SD = Standard Deviation.


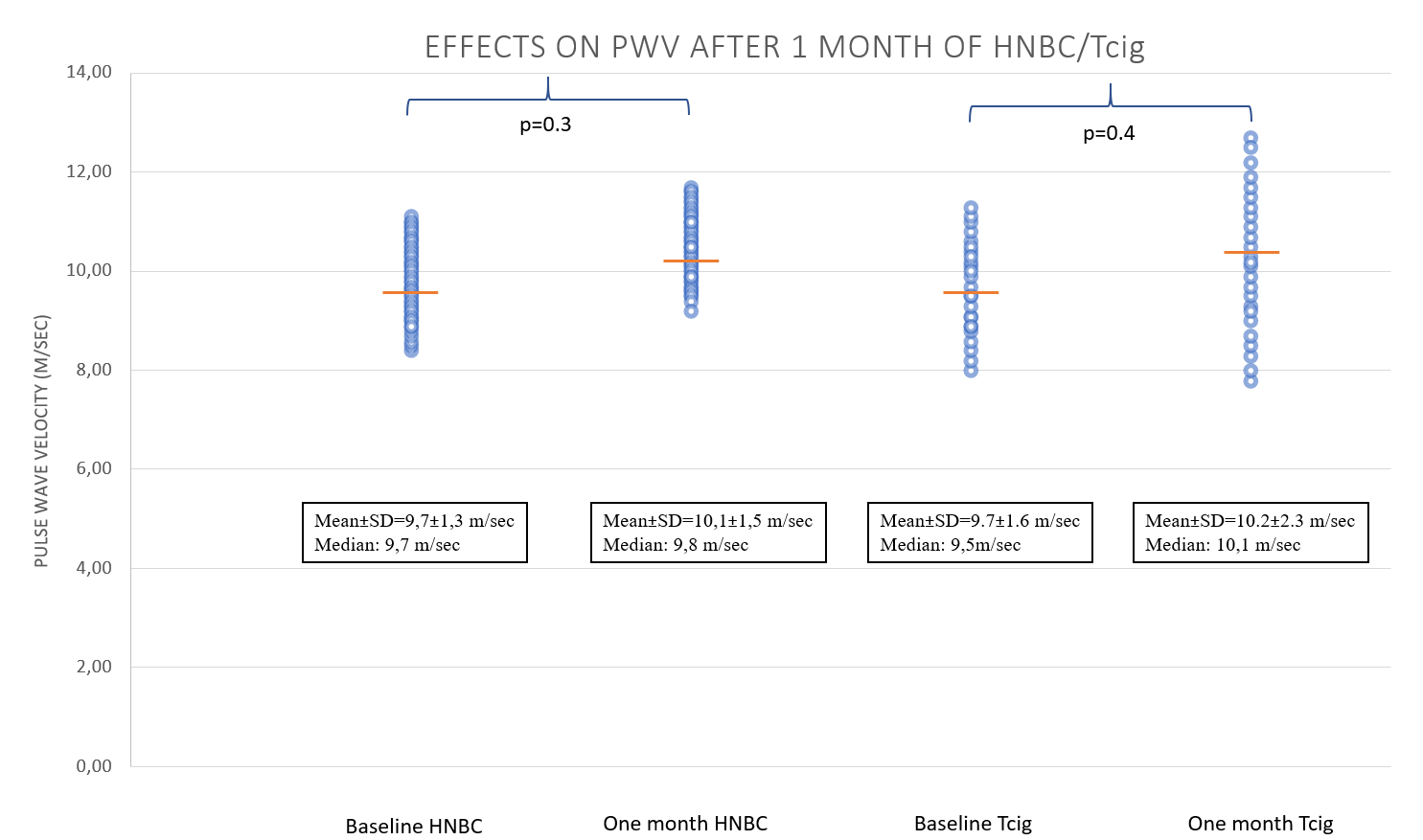


SUPPLEMETARY FIGURE 7. Scatterplot that represents PWV values (m/sec) of the study group and the control group during the chronic study. For the chronic phase, all participants of the acute phase (50 subjects), were instructed to replace Tcig smoking with HNBC puffing for 1 month and were compared with an external group of 25 Tcig smokers, before and after one month. Red line represents the mean value. In the box we report the mean ±SD and the median. PWV = carotid-femoral pulse wave velocity; HNBC = heat-not-burn cigarette; Tcig = tobacco cigarette; SD = Standard Deviation.


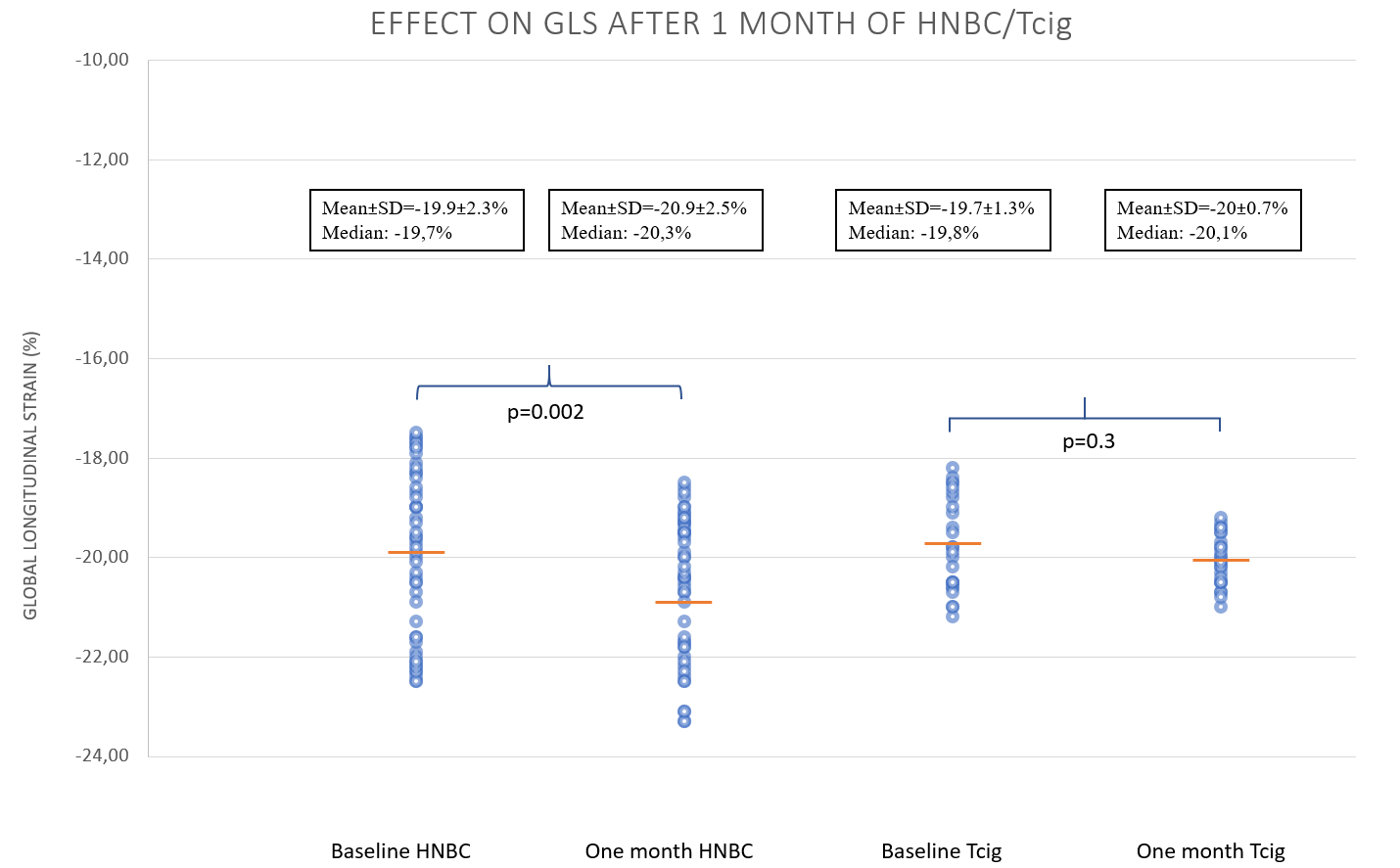


SUPPLEMETARY FIGURE 8. Scatterplot that represents GLS values (%) of the study group and the control group during the chronic study. For the chronic phase, all participants of the acute phase (50 subjects), were instructed to replace Tcig smoking with HNBC puffing for 1 month and were compared with an external group of 25 Tcig smokers, before and after one month. Red line represents the mean value. In the box we report the mean ±SD and the median. GLS = Global Longitudinal Strain; HNBC = heat-not-burn cigarette; Tcig = tobacco cigarette; SD = Standard Deviation.


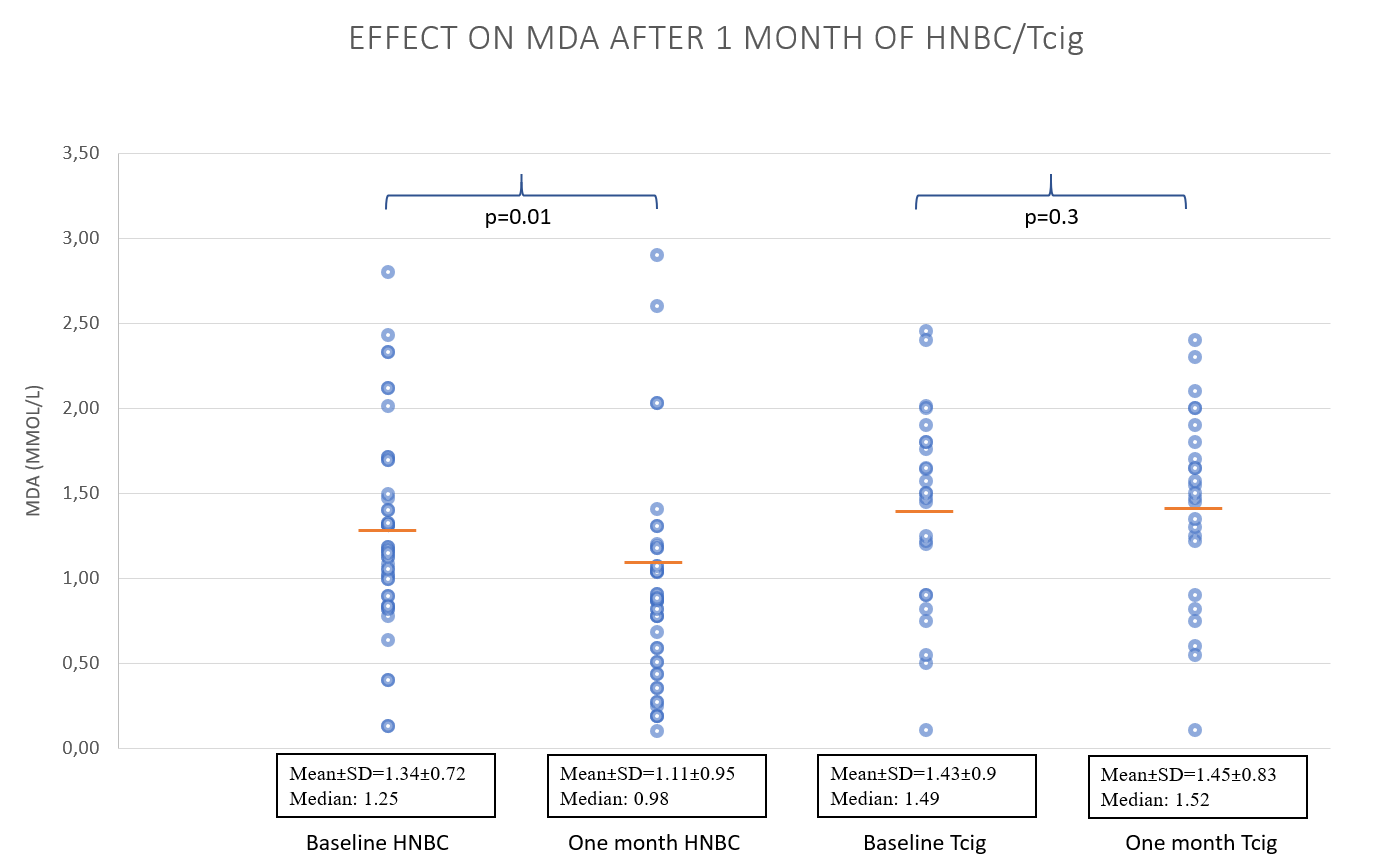


SUPPLEMETARY FIGURE 9. Scatterplot that represents MDA values (nmol/L) of the study group and the control group during the chronic study. For the chronic phase, all participants of the acute phase (50 subjects), were instructed to replace Tcig smoking with HNBC puffing for 1 month and were compared with an external group of 25 Tcig smokers, before and after one month. Red line represents the mean value. In the box we report the mean ±SD and the median. MDA = malondialdehyde; HNBC = heat-not-burn cigarette; Tcig = tobacco cigarette; SD = Standard Deviation.


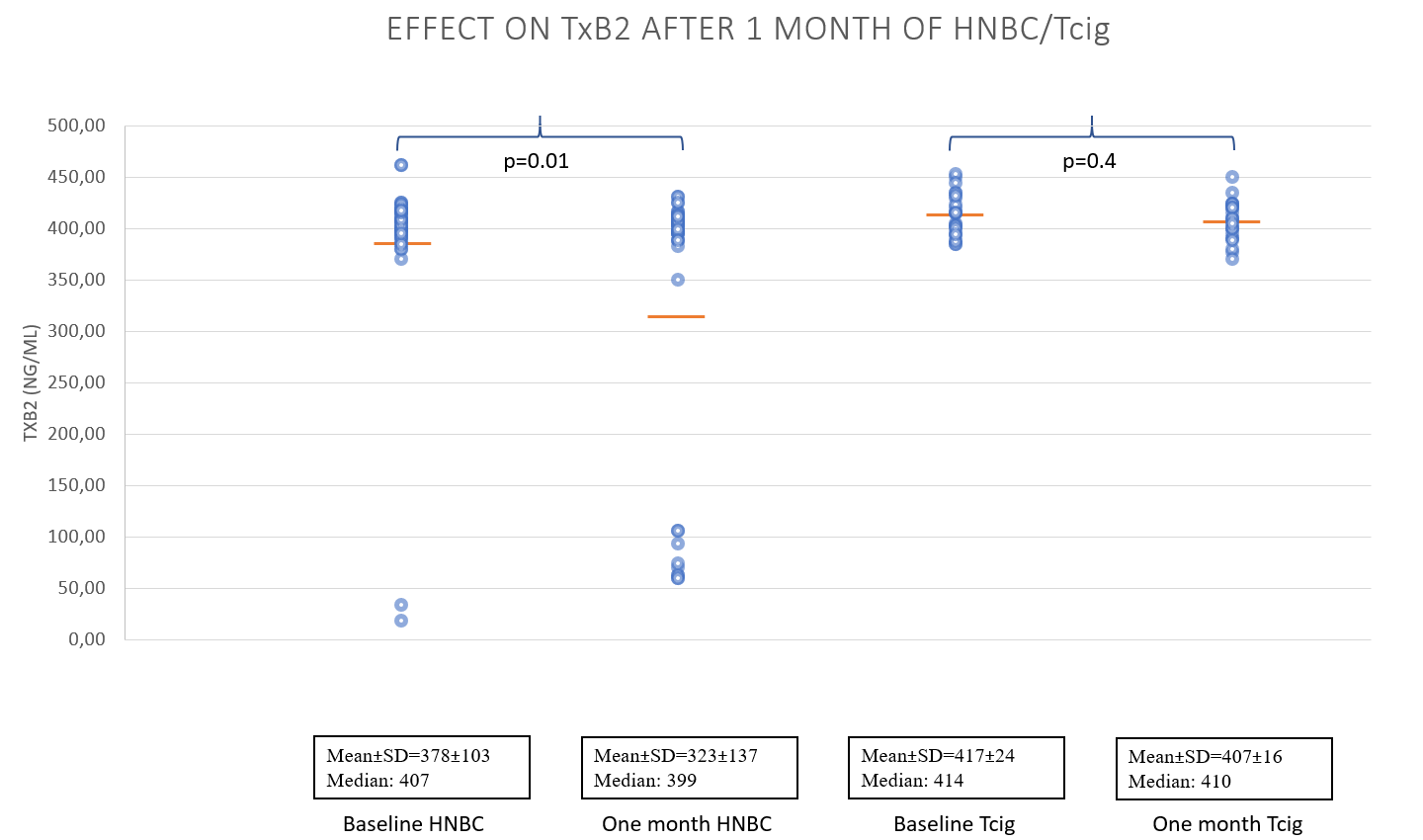


SUPPLEMETARY FIGURE 10. Scatterplot that represents TxB2 values (ng/mL) of the study group and the control group during the chronic study. For the chronic phase, all participants of the acute phase (50 subjects), were instructed to replace Tcig smoking with HNBC puffing for 1 month and were compared with an external group of 25 Tcig smokers, before and after one month. Red line represents the mean value. In the box we report the mean ±SD and the median. TxB2 = thromboxane B2; HNBC = heat-not-burn cigarette; Tcig = tobacco cigarette; SD = Standard Deviation.


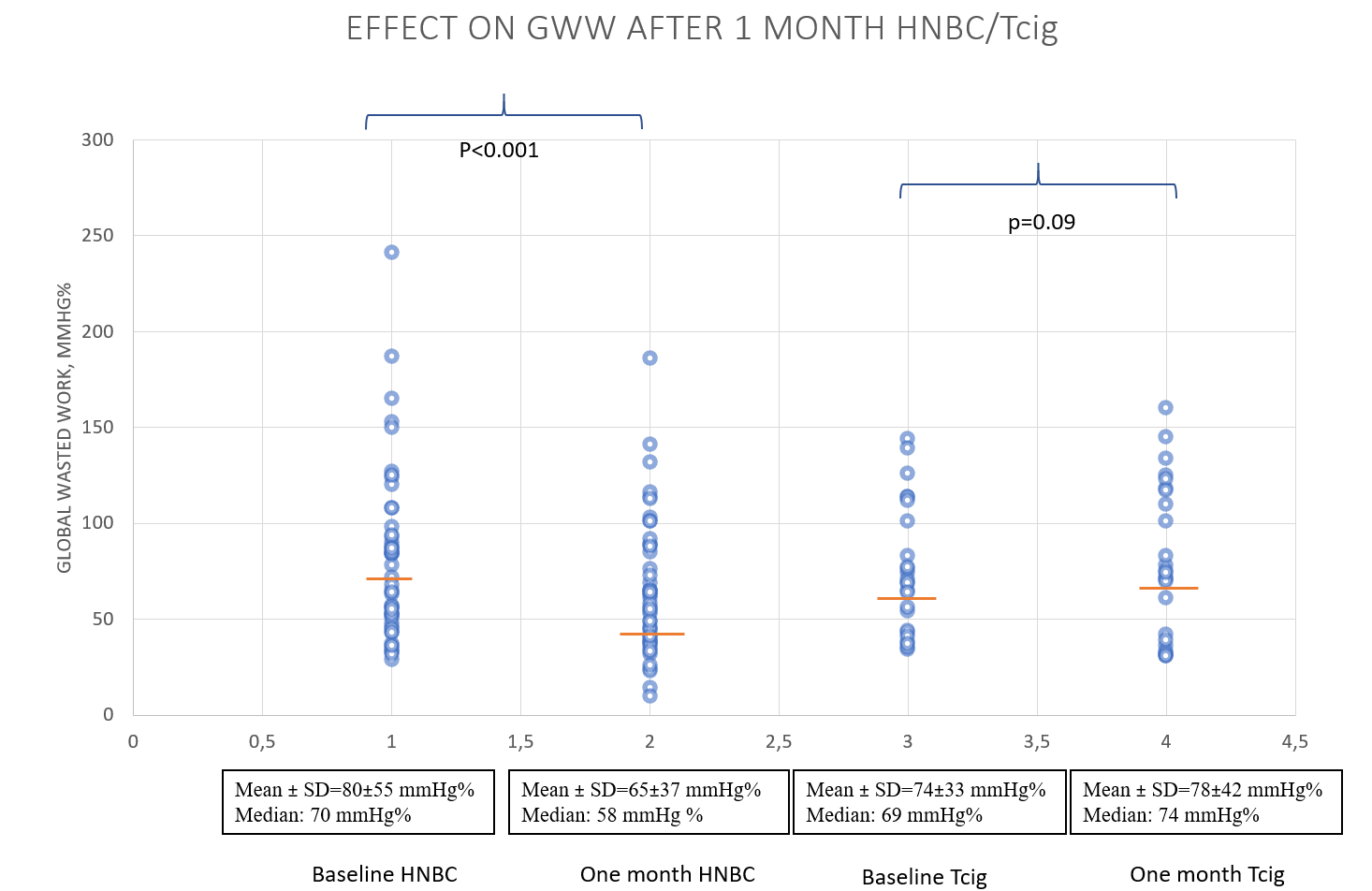


SUPPLEMETARY FIGURE 11. Scatterplot that represents GWW values of the study group and the control group during the chronic study. For the chronic phase, all participants of the acute phase (50 subjects), were instructed to replace Tcig smoking with HNBC puffing for 1 month and were compared with an external group of 25 Tcig smokers, before and after one month. Red line represents the mean value. In the box we report the mean ±SD and the median. GWW = Global Wasted Work; HNBC = heat-not-burn cigarette; Tcig = tobacco cigarette; SD = Standard Deviation.
